# Novel Antimicrobial Peptide from Temporin L in The Treatment of *Staphylococcus pseudintermedius* and *Malassezia pachydermatis* in Polymicrobial Inter-Kingdom Infection

**DOI:** 10.3390/antibiotics9090530

**Published:** 2020-08-22

**Authors:** Rosa Bellavita, Adriana Vollaro, Maria Rosaria Catania, Francesco Merlino, Luisa De Martino, Francesca Paola Nocera, Marina DellaGreca, Francesca Lembo, Paolo Grieco, Elisabetta Buommino

**Affiliations:** 1 Department of Pharmacy, University of Naples Federico II, Via Montesano 49, 80131 Naples, Italy; rosa.bellavita@unina.it (R.B.); francesco.merlino@unina.it (F.M.); frlembo@unina.it (F.L.); paolo.grieco@unina.it (P.G.); elisabetta.buommino@unina.it (E.B.); 2 Department of Molecular Medicine and Medical Biotechnology, University of Naples Federico II, Via S. Pansini 5, 80131 Naples, Italy; vollaroadriana@libero.it; 3 Department of Veterinary Medicine and Animal Production, University of Naples “Federico II”, Via Federico Delpino 1, 80137 Naples, Italy; ldemarti@unina.it (L.D.M.); francescapaola.nocera@unina.it (F.P.N.); 4 Department of Chemical Sciences, University Federico II of Naples, Complesso Universitario Monte S. Angelo, via Cinthia, 4, 80126 Naples, Italy; dellagre@unina.it

**Keywords:** *Staphylococcus pseudintermedius*, *Malassezia pachydermatis*, pets, interkingdom polymicrobial infections, antimicrobial peptides, Temporin L

## Abstract

Interkingdom polymicrobial diseases are caused by different microorganisms that colonize the same niche, as in the case of yeast-bacteria coinfections. The latter are difficult to treat due the absence of any common therapeutic target for their elimination, both in animals and humans. *Staphylococcus pseudintermedius* and *Malassezia pachydermatis* belong to distinct kingdoms. They can colonize the same skin district or apparatus being the causative agents of fastidious pet animals’ pathologies. Here we analysed the antimicrobial properties of a panel of 11 peptides, derived from temporin L, against *Malassezia pachydermatis.* Only peptide **8** showed the best mycocidal activity at 6.25 μM. Prolonged application of peptide **8** did not cause *M. pachydermatis* drug-resistance. Peptide **8** was also able to inhibit the growth of *Staphylococcus pseudintermedius*, regardless of methicillin resistance, at 1.56 μM for methicillin-susceptible *S. pseudintermedius* (MSSP) and 6.25 μM for methicillin-resistant *S. pseudintermedius* (MRSP). Of interest, peptide **8** increased the susceptibility of MRSP to oxacillin. Oxacillin MIC value reduction was of about eight times when used in combination with peptide **8**. Finally, the compound affected the vitality of bacteria embedded in *S. pseudintermedius* biofilm. In conclusion, peptide **8** might represent a valid therapeutic alternative in the treatment of interkingdom polymicrobial infections, also in the presence of methicillin-resistant bacteria.

## 1. Introduction

Polymicrobial inter-kingdom infections represent a serious problem in clinical practice [1]. The copresence of bacteria and fungi at the site of infection can decrease antimicrobial efficacy and the administration of a combined mixture of antimicrobials is necessary. *Malassezia pachydermatis* is part of the normal microbiota of the skin and ear canal of dogs and cats where it causes dermatitis. It is also the most common microorganism isolated from canine otitis externa [2]. The latter is a non-lethal chronic disease that troubles both dogs and owners for a long period. *M. pachydermatis* can be isolated from healthy and diseased human skin, and it is also the causative agent of nosocomial infections in neonates suggesting the transmission from pet animals [3,4,5]. Due to the recurrence of yeast infections, routine antifungal administration in pets may induce acquired resistance, leading to treatment failure. This is also because a common therapy with azole can be prolonged until 4 weeks favouring the acquisition of mutation and the selection of resistant strains. *Staphylococcus pseudintermedius* belongs to the normal flora of healthy dogs [6]. It is also considered a major pathogen in dogs, typically involved in skin and ear, urinary tract, and postoperative infections [7]. Temporins, isolated from the skin of *Rana temporaria*, represent one of the largest families of AMPs [8,9,10]. They are primarily active against Gram-positive bacteria and do not show hemolytic activity, except for isoform L (temporin L, TL) (Phe-Val-Gln-Trp-Phe-Ser-Lys-Phe-Leu-Gly-Arg-Ile-Leu-NH_2_) [11]. In the past, a promising molecule, named [Pro^3^, dLeu^9^] (**1**), Phe-Val-Pro-Trp-Phe-Ser-Lys-Phe-DLeu-Gly-Arg-Ile-Leu-NH_2_, was obtained and proved to be an efficient antimicrobial agent, devoid of cytolytic effects in vitro [12]. Peptide **1** was considered as a lead for a subsequent structure-activity relationship (SAR) study, focused on the Gly^10^, which was replaced by diverse amino acids with the aim to improve the antimicrobial activity (named peptide **9** in Merlino et al.) [13]. Starting from these promising outcomes, we explored the antimicrobial activity of a compound library, recently realized by our group, on *M. pachydermatis* [13]. After a first screening of all compounds, only the most interesting was further evaluated for its antimicrobial activity on *S. pseudintermedius*, with the aim to discover novel agents effective against polymicrobial infections.

## 2. Results

### 2.1. Antimicrobial Susceptibility of M. pachydermatis

The minimum inhibitory concentration (MIC) values of tested compounds against *M. pachydermatis* are reported in Table 1. Peptide **8** showed the highest inhibitory properties with a MIC value of 6.25 μM. Peptides **7** and **10** inhibited the yeast growth at a MIC value of 12.5 μM, while the other compounds were not so effective. The minimum mycocidal concentration (MMC) of peptide **8**, causing ≥3 log_10_ reduction in colony count from the starting inoculum, was 6.25 μM. The MMC/MIC ratio of 1 indicated a mycocidal activity of peptide **8**.

### 2.2. Resistance Acquisition Tests to Peptide ***8***

To evaluate if the yeast acquired resistance to peptide **8** after a prolonged treatment, *M. pachydermatis* was subcultured through serial passaging in the presence of a sublethal peptide **8** concentration (3.12 μM). After 1 generation subculture, peptide **8** reduced cell growth, affecting only in part yeast cell vitality (Table 2); however, after 15 yeast subcultures (about 2 months treatment), *M. pachydermatis* was no more able to replicate in the presence of peptide **8**. 

### 2.3. Antimicrobial Susceptibility of S. pseudintermedius 

**Figure 1:**
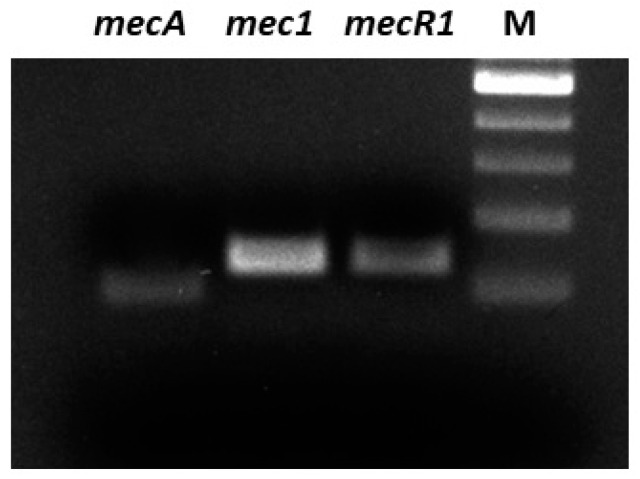
Detection of genes from mec operon in methicillin-resistant *S. pseudintermedius* (MRSP) strain. M: 100bp ladder marker (Biotech Rabbit).

Two strains of *S. pseudintermedius* were characterized for their pattern of antibiotic susceptibility as previously reported [14]. MRSP resulted oxacillin resistant and MSSP oxacillin susceptible. Both strains were screened for the presence of *mec*A, *mec*1, *mec*R1 genes by polymerase chain reaction (PCR). Only MRSPdemonstrated to possess the *mec*A operon (Figure 1).

Peptide **8** exhibited a significant MIC value at 1.56 μM for MSSP and 6.25 μM for MRSP (Table 3). As expected, oxacillin treated MRSP showed a MIC value forty times higher than MSSP. The minimum bactericidal concentration (MBC) resulted 3.12 μM for MSSP and 12.5 μM for MRSP. The MBC/MIC ratio values are reported in Table 3. 

To confirm the bactericidal or bacteriostatic activity of peptide 8 we performed the time kill assay at the MIC value (Table 4). Peptide **8** inhibited bacterial growth already at 1 h. After 6h treatment, a dramatic decrease in cell growth was observed. However, the number of both MSSP and MRSP cells increased 24 h after peptide **8** treatment. These results supported a bacteriostatic activity of peptide **8**.

### 2.4. Synergistic Study

The synergism between peptide **8** and oxacillin against MRSP was determined using the checkerboard technique. The highest synergistic interaction was obtained with the combination values of 1.56 μM peptide **8** (1/4 MIC) and 3.1 μM oxacillin (1/8 MIC). The FIC index, equal to 0.37, confirmed the synergistic effect of peptide **8** and oxacillin (Table 3).

### 2.5. Effect of Peptide ***8*** on Biofilm Formation 

**Figure 2:**
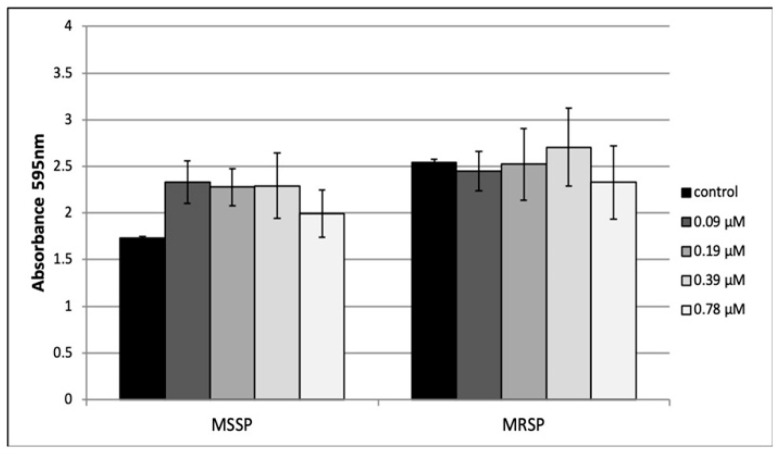
Effect of sub-MIC peptide **8** concentrations on MSSP and MRSP biofilm formation assessed by crystal violet assay. Experiments were performed in triplicate in three independent experiments.

The ability of peptide **8** to inhibit MSSP and MRSP biofilm formation was investigated by crystal violet (CV) assay. Peptide **8** was tested at sub-MIC concentrations ranging from 0.095 to 0.78 μM for 24 h. The highest concentration used (0.78 μM) did not affect planktonic growth. As shown in Figure 2 peptide **8** was ineffective on MSSP and MSRP biofilm formation at each tested concentration.

### 2.6. Effect of Peptide ***8*** on Mature Biofilm

**Figure 3:**
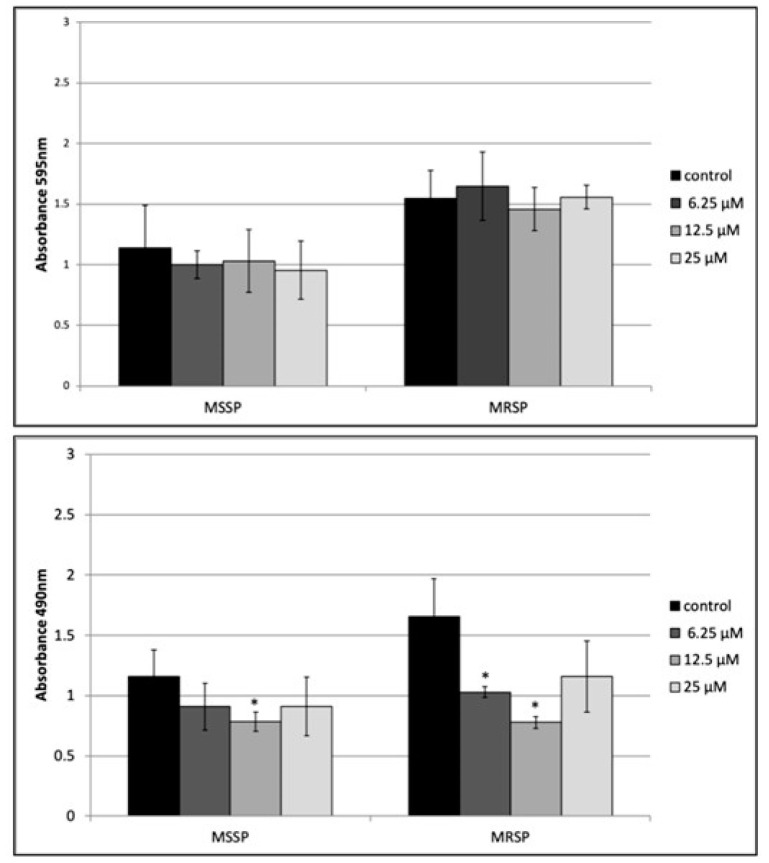
Peptide **8** activity on MSSP and MRSP mature biofilm assessed by crystal violet assay (upper panel) and XTT assay (bottom panel) Experiments were performed in triplicate in three independent experiments. * *p* < 0.05.

One-day-old-biofilms of both MSSP and MRSP strains were incubated with peptide **8** at 4× MIC, 2× MIC and 1× MIC for 24 h. CV results revealed that peptide **8** did not affect the biomass of both treated biofilms at concentrations up to 25 μM (Figure 3, upper panel). On the contrary, XTT assay clearly demonstrated that peptide **8** caused a significant decrease of both MSSP and MSRP biofilm viability (Figure 3, bottom panel). It was able to reduce biofilm viability of MRSP at 6.25 and 12.5 μM, by 38% and 52%, respectively, and 38% for MSSP at 12.5 μM. At 25 μM, we observed a reduced effect.

**Figure 4:**
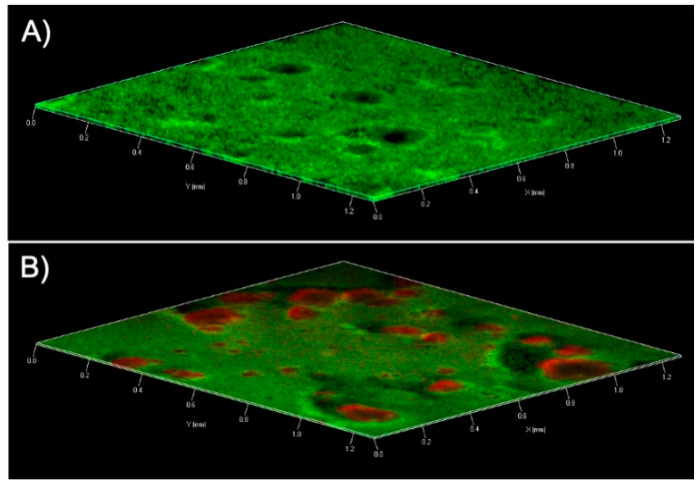
Peptide **8** effects on MRSP mature biofilm assessed by Confocal laser microscopy. (**A**) Untreated biofilm; (**B**) treated biofilm (32 μg/mL).

Finally, confocal microscopy images of treated biofilm showed some red zone representing cells within biofilm that were killed or damaged by peptide **8** (Figure 4). 

### 2.7. Influence of Peptide ***8*** on MecA Gene Expression

Quantification data obtained by RT-PCR were normalized to the reference gene for 16S rRNA. The results showed that peptide **8** did not regulate *mec*A gene expression, thus leading us to think the involvement of other mechanisms of action (data not shown). 

## 3. Discussion

The increase in antibiotic resistance is still a serious health problem, due to the indiscriminate use of antibiotics in animals and humans. The situation has become more critical in the hospital settings due to ease with which genes of resistance can be transmitted between multidrug-resistant strains and wild-type bacteria. In this context, polymicrobial infections represent an added problem since the presence of microorganisms, belonging to different reigns or different genus, forced to the use of a mix of antimicrobials to eradicate the infection. *S. pseudintermedius* and *M. pachydermatis* are often associated with otitis externa in dogs. Since antimicrobial resistance has been documented in both species with increasing frequency, there is an urgent need to discover new antimicrobial able to contrast the growth of species associated with polymicrobial infections. We showed for the first time the activity of a compound derived from a SAR study on the TL analogue [Pro^3^, dLeu^9^], that reduces the growth of both *M. Pachydermatis* and *S. pseudintermedius* (MSSP and MRSP). Of the 11 peptides tested only peptide **8** showed the best antimicrobial activity, compared to parent peptide **1**.

Azole drugs represent the first choice to treat *Malassezia*-related infections. It is well known that routine antifungal administration may induce acquired resistance [15]. Peptide **8** was further investigated to verify if prolonged treatment might induce resistance in the yeast. *M. pachydermatis* did not acquire resistance against the peptide after 2 months of repeated sub lethal concentration treatment. Thus, a prolonged application of peptide **8** can be resolutive for yeast infection, without causing drug-resistance. By considering the good results obtained until here, we were prompted to investigate the antimicrobial activity of peptide **8** on both MSSP and MRSP strains. Our results showed that peptide **8** inhibited the growth of both *S. pseudintermedius* strains (MSSP and MRSP). Previous results already reported the anticandidal and antibacterial activities of peptide **8** [13]. Peptide **8** did not show hemolytic activity on circulating blood cells and demonstrated low cytotoxicity on human keratinocytes at MICs. In addition, a recent work demonstrated that peptide **8** displayed a potent anti-inflammatory activity in in vivo model of acute inflammation [16]. A study by our group, focused on the interaction of peptide **8** with bacterial membrane, has shown that it displayed a membrane-perturbing activity on both Gram-positive and Gram-negative strains. Peptide **8** is monomeric with a random coil conformation in aqueous solution (unpublished results); on the contrary, it shows a significant ability to aggregate in membrane mimetic environments, altering the membrane fluidity and creating pores both in Gram-positive and Gram-negative membranes. We hypothesize a similar mechanism of action of peptide **8** on MSSP and MRSP. The interaction of the peptide with the membrane at the MBC could compromise the integrity of the microbial cell’s barrier structures as generally reported for the AMPs [17]. Due to the nature of peptides, they could interact with acidic negatively charged phospholipids in the microorganism membranes, leading to increased permeability and loss of integrity, with the occurrence of cell death. In our case, at MIC value maybe the peptide does not reach the critical bactericidal concentration on all cell membranes causing the death of some bacteria (highest cell membrane accumulation) and only cell growth arrest of others (low accumulation). This might explain why in the time kill assay we observed a drastic decrease of cell number at the early hours of treatment and a partial recovery of cell growth 24 h after. In the case of *M. pachydermatis* we can only suppose a similar mechanism, since we have not yet performed studies mimicking the yeast cell membrane.

In order to safeguard the future effectiveness of critically important antibiotics, it is possible to use old drugs in synergic association with a new antimicrobial, able to potentiate or restore their efficacy. We demonstrated that peptide **8** increased the susceptibility of MRSP to oxacillin with an oxacillin MIC reduction of about eight times (oxacillin alone versus oxacillin in combination). Synergy observed in the combination of antimicrobial peptides with conventional antibiotics is generally attributed to the ability of AMP to enhance the cell membrane permeability, thus allowing the entry of antibiotics [18]. Extended/random coil peptides tend to fold into amphipathic structures when in contact with a biological membrane [19]. Their activity is correlated to membrane leakage, but also interaction with intracellular targets causing nucleic acid or cell wall synthesis inhibition. Studies about AMPs synergy with β-lactams are reported, but the exact mechanism of resistance attenuation is yet unknown [20]. To understand how peptide **8** improved the activity of oxacillin, we investigated if it might modulate *mecA* gene expression. Unfortunately, we did not demonstrate gene regulation. We cannot exclude that at MIC value the interaction with other intracellular targets occurs, besides those that cause membrane perturbation. Chronic biofilm-related infections pose a challenge for antimicrobials. Peptide **8** caused a significant decrease of MSSP and MRSP biofilm viability compared to the corresponding untreated control. Consequently, even though we did not observe a reduction in the biofilm formation the molecule was able to kill bacteria embedded in the biofilm polymers. In this regard, TL (precursor of peptide **8**) recently proved to be an effective agent able to affect *P. aeruginosa* PAO1 and methicillin-resistant *S. aureus* (MRSA) biofilms [21]. Similarly, peptide **8** due to positive charges distributed in the peptide sequence, might electrostatically interact with the extracellular matrix of biofilm, thus facilitating transition through this environment and improving its antimicrobial activity. 

## 4. Materials and Methods 

### 4.1. Materials

All *N^α^*-Fmoc-protected amino acids were purchased from GL Biochem Ltd. (Shanghai, China). Coupling reagents, such as *N*,*N*,*N*′,*N*′-tetramethyl-*O*-(1*H*-benzotriazol-1-yl) uranium hexafluorophosphate (HBTU) and 1-hydroxybenzotriazole (HOBt), as well as the Rink amide resin used, were commercially obtained by GL Biochem Ltd. (Shanghai, China). Unconventional *N^α^*-Fmoc-amino acids, such as Fmoc-l-Hyp(*t*Bu) and Fmoc-d-Hyp(*t*Bu) were purchased from Sigma-Aldrich and Fmoc-Aic was acquired by Fluorochem. (*N*,*N*-diisopropylethylamine (DIEA), piperidine and trifluoroacetic acid (TFA) were purchased from Iris-Biotech GMBH. Moreover, peptide synthesis solvents and reagents, such as *N*,*N*-dimethylformamide (DMF), dichloromethane (DCM), diethyl ether (Et_2_O), water and acetonitrile (MeCN) for HPLC, were reagent grade acquired from commercial sources (Sigma-Aldrich and VWR) and used without further purification. Unless otherwise stated, all the other reagents were from Carlo Erba (Milan, Italy).

### 4.2. Peptides Synthesis

The synthesis of peptides was performed by using the ultrasound-assisted solid-phase peptide strategy (US-SPPS) combined with the orthogonal Fmoc/*t*Bu chemistry, as elsewhere reported [22]. In particular, each peptide was assembled on a Rink amide resin (0.1 mmol from 0.64 mmol/g of loading substitution), as solid support. Fmoc-deprotections were performed treating the resin with a 20% piperidine solution in DMF, 0.5 + 1 min, whereas each coupling was carried out by using Fmoc-aa (2 equiv), HBTU/HOBt (2 equiv) and DIEA (4 equiv), 5 min. Finally, peptides were purified and characterized by RP-HPLC using linear gradients of MeCN (0.1% TFA) in water (0.1% TFA), from 10 to 90% over 20 min (Table 5). 

### 4.3. Microbial Strains and Culture

*M. pachydermatis* was previously isolated and characterized as reported [23]. The yeast was cultured onto Sabouraud dextrose agar with chloramphenicol (Oxoid Ltd., London, UK) at 30 °C. Veterinary clinical strains of *S. pseudintermedius* were isolated from auricular swabs of dogs suffering from otitis externa and processed at the Microbiology Laboratory of the Department of Veterinary Medicine and Animal Production, University of Naples “Federico II” (Italy). *S. pseudintermedius* strains were plated on blood agar base supplemented with 5% sheep blood and on mannitol-salt agar, and incubated aerobically at 37 °C for 24–48 h. The strains were identified by matrix-assisted laser desorption ionization-time of flight mass spectrometry (MALDI-TOF MS) (Bruker Daltonics, Macerata, Italy). The antimicrobial susceptibility patterns of Staphylococci strains were determined by disk diffusion test on Mueller–Hinton agar (Oxoid Ltd., London, UK). The inhibitory zone diameters obtained around the antibiotic disks were measured after incubation for 24 h at 37 °C and evaluated according to the Clinical and Laboratory Standards Institute (CLSI 2018) [24].

### 4.4. Molecular Analysis

Genomic DNA extraction was performed by using GenUp Bacteria gDNA kit (BiotechRabbit, Berlin, Germany) according to the manufacturer’s instructions. All the isolates were tested for genes of the *mec* operon using the polymerase chain reaction (PCR). One µL of DNA was amplified in a reaction mixture containing 10 mM Tris–HCl (pH = 8.3), 1.5 mM MgCl_2_, 50 mM KCl, 10 μM dNTP and 10 μM forward and reverse primers (*mecA*, *mec1* and *mecR1*) and 2.5 U of Taq DNA polymerase (BiotechRabbit, Berlin, Germany) in a final volume of 25 µL. The cycling conditions are reported in Table 6.

The reaction was carried out in a DNA thermal cycler (MyCycler, Bio-Rad, Hercules, CA, USA). The PCR products were analysed by electrophoresis on 1.8% agarose gel in TBE and analysed on a Gel Doc EZ System (Bio-Rad, Hercules, CA, USA). RNA extraction was performed by using GenUp Total RNA kit (BiotechRabbit, Berlin, Germany) according to the manufacturer’s instructions. Five hundred nanograms of total cellular RNA were reverse-transcribed (RevertUP II Reverse Transcriptase, BiotechRabbit, Berlin, Germany) into cDNA using random hexamer primers (Random hexamer, Roche Diagnostics, Mannheim, Germany) at 48 °C for 60 min according to the manufacturer’s instructions. RT-PCR was carried out using 2 μL of cDNA, as reported above. Quantification data were normalized to the reference gene for 16S rRNA gene and analyzed by Image Lab software 5.2.1 (Bio-Rad, Hercules, CA, USA).

### 4.5. Resistance Acquisition Tests to Peptide ***8***

To evaluate if the yeast acquired resistance to the drug, after a continued treatment, *M. pachydermatis* was subcultured with a sub lethal concentration of peptide **8** (1/2 MIC). Briefly, a final 3.12 μM peptide **8** concentration was added to yeast inoculum suspension equivalent to 1–3 × 10^6^ CFU/mL in Sabouraud dextrose broth (SB) and incubated 72 h at 30 °C (inoculum 1). After this period, optical density was measured and a new yeast subculture with 3.12 μM of peptide **8** was prepared starting from inoculum 1 (inoculum 2). Three days after, optical density was again recorded and a new inoculum in presence of peptide **8** prepared. This was repeated until evident yeast death was observed.

### 4.6. Antimicrobial Activity Assay

MIC of all compounds was determined in Sabouraud Dextrose broth with 1% tween 80 (SB) medium, for *M. pachydermatis*, and Mueller–Hinton broth (MH), for *S. pseudintermedius*, by the broth microdilution assay (BMD) in 96-well microtiter plates, as previously reported [25]. Specifically, SB containing 1% Tween 80 was used instead of RPMI 1640 medium. All compounds were dissolved in ethanol and ketoconazole in dimethyl sulfoxide (DMSO). The solutions were serially diluted in SB and MH two-fold from 0.19 to 50 μM. *M. pachydermatis* suspension was prepared from 3-day-old colonies grown on Sabouraud agar at 30 °C. The final concentration of the yeast inoculum suspensions was equivalent to 1–3 × 10^3^ CFU/mL. Bacterial suspensions were diluted to yield an optical density (OD) around 0.5 at 595 nm and further diluted to a final concentration of 1 × 10^6^ CFU/mL. The compounds were added to the microbial suspension in each well yielding a final cell concentration of 5 × 10^5^ CFU/mL. Positive controls included oxacillin 5 μM (2 μg/mL) and vancomycin 1.4 μM (2 μg/mL) for *S. pseudintermedius* or ketoconazole 0.2 μM (0.125 μg/mL) for *M. pachydermatis*. Negative control wells were set to contain bacteria in MH or yeasts in SB plus the amount of ethanol used to dilute each compound. Medium turbidity was measured by a microtiter plate reader (Tecan, Milan, Italy) at 595 nm after 24 h for *S. pseudintermedius* and 48 h for *M. pachydermatis*. Absorbance was proportional to microbial growth. The MIC was defined as the lowest concentration of drug that caused a prominent decrease (≥90% inhibition) in visible growth relative to that of the growth control. MBC and MMC were defined as the concentration that caused ≥3log_10_ reduction in colony count from the starting inoculum plated on agar medium, incubated for 24 h at 37 °C and 72 h at 30 °C, respectively.

### 4.7. Killing Rate

Time kill assay was carried out as previously described [26]. Bacterial suspension (10^5^ CFU/mL) was added to microplates along with peptide **8** at 6.25 μM MIC concentration. Plates were incubated at 37 °C on an orbital shaker at 120 rpm. Viability assessments were performed at 0, 2, 4, 6 and 24 h by plating 0.01 mL undiluted and 10-fold serially diluted samples onto Mueller–Hinton plates in triplicate. After overnight incubation at 37 °C, bacterial colonies were counted and compared with counts from control cultures.

### 4.8. Checkerboard Method

The interaction between peptide **8** and oxacillin against MRSP was evaluated by the checkerboard method. Briefly, twofold serial dilutions of oxacillin distributed in horizontal rows of 96-well microtiter plate were cross-diluted vertically by twofold serial dilutions of peptide **8** to at least double the MIC. The peptide **8** tested concentration ranging from 0.19 to 12.5 μM and oxacillin from 1.25 to 25 μM (0.5 to 10 μg/mL). Microtiter plates were inoculated with bacteria at an approximate concentration of 10^5^ CFU/mL and incubated at 37 °C for 24 h. MIC values of the combinations were determined as the lowest concentrations that completely inhibited bacterial growth, recorded as optical density at 595 nm. To evaluate the effect of the combination treatment, the fractional inhibitory concentration (FIC) index for each combination was calculated as follows: FIC index = FIC of peptide **8** + FIC of oxacillin, where FIC of peptide **8** (or oxacillin) was defined as the ratio of MIC of peptide **8** (or oxacillin) in combination and MIC of peptide **8** (or oxacillin) alone. The FIC index values were interpreted as follows: ≤0.5, synergistic; >0.5 to ≤1.0, additive; >1.0 to ≤2.0, indifferent; and >2.0, antagonistic effects [27].

### 4.9. Effect of Peptide ***8*** on Biofilm Formation

Anti-biofilm activity of peptide **8** was examined by the crystal violet assay previously described with minor modifications [28]. Microtiter plates were inoculated with bacteria at a final density of 10^6^ CFU/mL and treated with peptide **8** ranging from 0.095 to 0.78 μM. Control cells were grown in the absence of peptide **8**. After 24 h incubation at 37 °C, the supernatant was gently removed, and the wells were rinsed twice with PBS. The amount of biofilm formed in the wells was measured by crystal violet staining and the absorbance of the solution was measured at 595 nm.

### 4.10. Effect of Peptide ***8*** on Mature Biofilm Biomass

Biofilms were allowed to form in each well of a 96-well microtiter plate, as described by Stepanovic [29]. After 24 h, planktonic cells were aspirated, and the plates were washed with 200 μL of PBS. Cells biofilms were exposed to 200 μL of peptide **8** at the final concentration ranging from 6.25 to 25 μM and the plate was further incubated for 24 h at 37 °C. Positive controls were non-treated cells incubated with 200 μL of medium broth. At the end of the experiment crystal violet-staining was performed to assess biofilm mass.

### 4.11. Quantitation of Metabolic Activity of Mature Biofilm by XTT Assay

The metabolic activity of MSSP and MRSP mature biofilms was quantified by the XTT [2,3-bis(2-methyloxy-4-nitro-5-sulfophenyl)-2H-tetrazolium-5-carboxanilide] (Roche Diagnostics, Germany) reduction assay. The assay was conducted as previously described with some modifications [30]. XTT (150 µL) was added to biofilms in each well and the plates were incubated for 40 min at 37 °C in the dark. The reduction of the tetrazolium salt by cellular dehydrogenase into orange formazan dye was photometrically measured at 490 nm. The medium was set as negative control. Viability values for each well were compared to controls.

### 4.12. Confocal Laser Scanning Microscopy (CLSM)

CLSM was used to confirm the effect of peptide **8** on mature biofilm respect the controls. MSRP were grown in chambered cover glass (μ Slide 4 well; ibidi GmbH, Germany) in a static condition for 24 h. Peptide **8** was added on a 1-day-old biofilm at 12.5 μM. Bacterial suspensions incubated with medium alone were used as a positive control. After 24 h, biofilms were rinsed with PBS and stained by using a LIVE/DEAD^®^ BacLight Bacteria Viability stains (Life Technologies, Italy). After the staining, the images were observed using a LSM 700 inverted confocal laser-scanning microscope (Zeiss, Italy). The areas were scanned using a 10X objective lens with the signal recorded in the green channel for Syto9 (excitation 488 nm, emission 500–525 nm) and in red channel for PI (excitation 500–550 nm, emission 610–650 nm).

## 5. Conclusions

Here, we demonstrated the antimicrobial activity of a new peptide able to contrast the growth of both *S. pseudintermedius* and *M. pachydermatis*. Peptide **8** did not cause yeast drug resistance and increased the susceptibility of oxacillin against MRSP, two aspects that make a new compound quite interesting. Consequently, the use of peptide **8** may provide novel avenues of possible therapeutic strategies to combat inter-kingdom infections, particularly otitis externa in dogs and cats, but also antimicrobial agents for topical treatment, such as wound infections in humans caused by zoonotic microorganisms. In addition, peptide **8** can be used in combination with conventional drugs to overcome the antibiotic resistance.

## Figures and Tables

**Table 1:** In vitro antifungal activity. Minimum inhibitory concentration (MIC), minimum mycocidal concentration (MMC) and MMC/MIC ratios for compounds (Cmpds) evaluated against *M. pachydermatis.*

Cmpds	MIC Values (μM)	MMC Values (μM)	MMC/MIC Ratio
**1**	25	100	4
**2**	25	100	4
**3**	25	100	4
**4**	25	100	4
**5**	25	100	4
**6**	25	100	4
**7**	12.5	25	2
**8**	6.25	6.25	1
**9**	25	100	4
**10**	12.5	25	2
**11**	25	100	4

**Table 2:** Resistance acquisition tests to 3.12 μM of cmpd **8**. Each experiment is the result of three independent experiments performed in triplicate.

Strains	Log10 CFU/Ml After 1 Generation Subculture Including TL48	Log10 CFU/Ml After 15 Generation Subcultures Including TL48
*M. pachydermatis* Untreated	8.10 ± 0.21	8.26 ± 0.29
*M. pachydermatis* + Cmpd 8	5.80 ± 0.25	2.10 ± 0.12

**Table 3:** MIC, minimum bactericidal concentration (MBC), MBC/MIC ratio, and FIC_index_ values (μM) of peptide **8** on selected bacterial strains. N.d. = not determined. Each experiment is the result of three independent experiments performed in triplicate.

Strains	MIC	MBC	Oxacillin	Vancomycin	MBC/MIC Ratio	FIC_index_
MSSP	1.56	3.12	<5	<1.4	2	n.d.
MRSP	6.25	12.5	25	<1.4	2	0.37

**Table 4:** Time-kill assay of peptide **8** against methicillin-susceptible *S. pseudintermedius* (MSSP) and MRSP. Each experiment is the result of three independent experiments performed in triplicate.

Strains	Log_10_CFU/mL					
	0 h	1 h	2 h	4 h	6 h	24 h
MSSP untreated	5.50 ± 0.21	5.74 ± 0.21	5.80 ± 0.25	5.87 ± 0.19	7.10 ± 0.31	9.8 ± 0.31
MSSP+TL48	5.45 ± 0.25	0.60 ± 0.12	0.58 ± 0.32	0.89 ± 0.15	0.46 ± 0.15	4.97 ± 0.30
MRSP untreated	5.30 ± 0.21	5.37 ± 0.32	5.84 ± 0.28	5.91 ± 0.29	6.85 ± 0.25	9.36 ± 0.25
MRS+TL48	5.30 ± 0.18	2.47 ± 0.18	2.38 ± 0.19	1.30 ± 0.21	0.77 ± 0.12	4.86 ± 0.28

**Table 5:** Sequences of compounds used in our study reported in Merlino et al. [13].

Cmpd	Sequence
**1**	F V P W F S K F l **Gly^10^** R I L
**2**	F V P W F S K F l **Pro^10^** R I L
**3**	F V P W F S K F l **dPro^10^** R I L
**4**	F V P W F S K F l **Hyp^10^**R I L
**5**	F V P W F S K F l **dHyp^10^** R I L
**6**	F V P W F S K F l **dNle^10^**R I L
**7**	F V P W F S K F l **Lys^10^**R I L
**8**	F V P W F S K F l **dLys^10^**R I L
**9**	F V P W F S K F l **Trp^10^**R I L
**10**	F V P W F S K F l **dTrp^10^**R I L
**11**	F V P W F S K F l **Aic^10^**R I L

The Gly^10^ and the diverse amino acids with which it was replaced, are indicated in bold.

**Table 6:** Staphylococcal sense and antisense primers sequences and expected polymerase chain reaction (PCR) products (bp).

Gene	Sense and Antisense Sequences	Conditions	Bp
*mecA*	5′-TCCACCCTCAAACAGGTGAA-3′ 5′-TGGAACTTGTTGAGCAGAGGT-3′	95 °C for 5′ 94 °C 30″, 55°C 4″s, 72 °C 30″ for 33 cycles 72 °C for 7′	139
*mecI*	5′-TCATCTGCAGAATGGGAAGTT-3′ 5′-TTGGACTCCAGTCCTTTTGC-3′		103
*mecR1*	5′-AGCACCGTTACTATCTGCACA-3′ 5′-AGAATAAGCTTGCTCCCGTTCA-3′		142
rRNA16S	5′-CGGTCCAGACTCCTACGGGAGGCAGCA-3′ 5′-GCGTGGACTACCAGGGTATCTAATCC-3′		450
